# Viral diversity and clonal evolution from unphased genomic data

**DOI:** 10.1186/1471-2164-15-S6-S17

**Published:** 2014-10-17

**Authors:** Hossein Khiabanian, Zachary Carpenter, Jeffrey Kugelman, Joseph Chan, Vladimir Trifonov, Elyse Nagle, Travis Warren, Patrick Iversen, Sina Bavari, Gustavo Palacios, Raul Rabadan

**Affiliations:** 1 Department of Systems Biology and Department of Biomedical Informatics, Columbia University College of Physicians and Surgeons, New York, New York, USA; 2 Genomics Division, The U.S. Army Medical Research Institute of Infectious Diseases, Fort Detrick, Maryland, USA; 3 Discovery Unit, Sarepta Therapeutics, Corvallis, Oregon, USA

**Keywords:** Clonal evolution, Evolutionary dynamics, Viral genomic diversity, Marburgvirus

## Abstract

**Background:**

Clonal expansion is a process in which a single organism reproduces asexually, giving rise to a diversifying population. It is pervasive in nature, from within-host pathogen evolution to emergent infectious disease outbreaks. Standard phylogenetic tools rely on full-length genomes of individual pathogens or population consensus sequences (phased genotypes).

Although high-throughput sequencing technologies are able to sample population diversity, the short sequence reads inherent to them preclude assessing whether two reads originate from the same clone (unphased genotypes). This obstacle severely limits the application of phylogenetic methods and investigation of within-host dynamics of acute infections using this rich data source.

**Methods:**

We introduce two measures of diversity to study the evolution of clonal populations using unphased genomic data, which eliminate the need to construct full-length genomes. Our method follows a maximum likelihood approach to estimate evolutionary rates and times to the most recent common ancestor, based on a relaxed molecular clock model; independent of a growth model. Deviations from neutral evolution indicate the presence of selection and bottleneck events.

**Results:**

We evaluated our methods *in silico *and then compared it against existing approaches with the well-characterized 2009 H1N1 influenza pandemic. We then applied our method to high-throughput genomic data from marburgvirus-infected non-human primates and inferred the time of infection and the intra-host evolutionary rate, and identified purifying selection in viral populations.

**Conclusions:**

Our method has the power to make use of minor variants present in less than 1% of the population and capture genomic diversification within days of infection, making it an ideal tool for the study of acute RNA viral infection dynamics.

## Background

A single rapidly evolving RNA virus can give rise to a swarm of related descendants [1]. Clonal expansions can be observed during an acute infection as pathogens replicate within a host [2,3] or in an outbreak of an emerging pathogen, when a novel virus propagates through a susceptible host population. A viral population diversifies as it expands, enabling the virus to explore larger sections of the fitness landscape [4]. Studying the dynamics of viral diversification can yield insight into when a host was originally infected, how fast a pathogen is evolving, and if specific genomic alterations are being selected for in a particular host or treatment regime.

Clonal populations founded by a single ancestor consist of individual organisms with highly similar, though not necessarily identical, genomes. The consensus genome is a constructed sequence representing the majority allele at each residue; hence, it may not truly exist in the viral population and fails to capture the whole mutant distribution in the sub-population structure. Viral diversity in acute infection has been previously studied through single genome amplification and combinations of RT-PCR and cloning [5,6]. These studies have utilized both phylogenetic techniques and exponential growth models to quantify viral evolution [5,7]. With advances in high-throughput sequencing technologies, studying viral genomic diversity and its role in inter- and intra-host evolution has become more feasible. Ultra-deep sequencing has been employed to investigate systems of chronic infections in which viral populations have reached sustained levels of diversity [8,9], as well as to investigate intra-host evolution of viral infections utilizing minor variants [10,11]. However, given estimated viral evolutionary rates of 10^-4 ^to 10^-6 ^substitutions/site/year, intra-host evolutionary dynamics during the first few days of an acute infection are dominated by very rare variants that only exist in less than 1% of the population [12]. Due to inherent limitation on the length of the reads produced by high-throughput sequencing technologies, standard phylogenetic algorithms and consensus-based methodologies fail as the coexistence of very rare polymorphisms in each individual viral clone cannot be determined. In other words, the mutations cannot be phased as the information of their linkage with respect to the viral genome is lost (Supplementary Fig. S1, in Additional file 1) [4,11,13].

In this manuscript, we introduce a method to study the dynamics of clonal evolutions without the need for phased data. Our methodology provides a means to estimate the starting time and evolutionary rates without assuming a model of growth. We validate our method both using a simulated clonal expansion and using genomic data from the 2009 H1N1 influenza pandemic. In the latter case, phylogenetic analyses using full-length genomes are treated as the gold standard, with which our evolutionary dynamic estimates strongly agree [14,15]. We then apply our method to genetic data where phase information is missing. Specifically, we infer the intra-host evolutionary dynamics of viral infections *in vivo*, using high-throughput, deep sequence data obtained from marburgvirus-infected non-human primates (NHP).

## Methods

**Figure 1:**
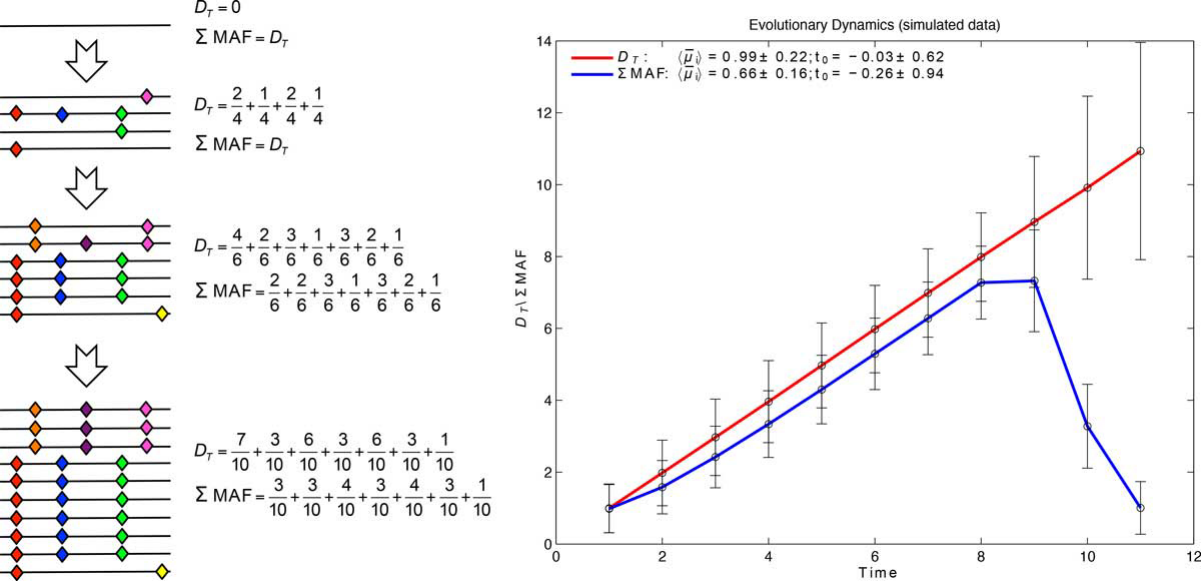
**Clonal expansions arise as asexual growth from a single clone**. **Left: **Phylogenetic algorithms compare the descendant clones across their genomes to reconstruct the evolutionary history. We, however, measure population diversity across the segregating sites, via summing their frequency, to estimate evolutionary properties. **Right: **The estimates for the simulated datasets. Estimates based on MAF represent the lower bound of those based on *D_T_*. The mean evolutionary rate, $\langle {\bar{\mu }}_{i}\rangle$, is given in 10^-4 ^substitutions/site per time point. The standard errors are derived from 95% confidence intervals via 1,000 simulated datasets.

**Measures of diversity**. If the genome of the expansion's initiating clone is known, the frequencies of the diverging alleles from the seed, as well as their genomic positions (segregating sites), are evident in its descendants. Therefore, we define total divergence, $D_{T}\left(t_{i}\right)=\sum_{s}x_{s}\left(t_{i}\right)$, where *x_s_*(*t_i_*) is the frequency of a diverging allele at time *t_i_*, positioned at segregating sites, *s*. Knowledge of the alleles present within the seeding clone is commonly unavailable. In lieu of this information, an approximated proxy for the initial seeding genome from the samples collected early in the expansion is often used. Even though some polymorphisms become fixed and some disappear from the population, *D_T_*, as a measure of divergence, will always increase with time (Figure 1).

To avoid approximating the genome of the initial seeding clone, we propose to estimate the genomic diversity at time *t_i _*with the sum of the minimal allele frequencies (MAF) at segregating sites. Minimal allele frequency can be best represented by one minus the frequency of the dominant allele at a segregating site. By definition, *x_s_*(*t_i_*) are always equal or larger than MAF; therefore, estimates based on sum of MAF represent the lower bound of those from *D_T_*. Strong differences between the two measures indicate selection or bottlenecks, as changes in *D_T _*measure time and divergence from the seed and the sum of MAF indicates variations in population diversity at a particular time.

**Mathematical framework.** Consider a clonal expansion with *N*(*t_i_*) clones at time *t_i_*, after a single initial clone began reproducing at time *t_0_*. Independent of a model of growth, we define ${\bar{\mu }}_{i}=\frac{1}{t_{i}-t_{0}}\overset{t_{i}}{\underset{t_{0}}{\int }}\mu \left(\tau \right)\text{d}\tau$ to be the average of evolutionary rates between time *t_i _*and *t_0_*. The average Hamming distance between any of these clones and the seed can be approximated by ${\bar{\mu }}_{i}l\left(t_{i}-t_{0}\right)$, where *l *is the size of the genome. Assuming that the *N*(*t_i_*) clones truly represent the frequencies of the segregating sites at time *t_i_*, $\langle d(t_{i})\rangle$, the expected distance of the descendants to the original clone, can be re-written as: $d\left(t_{i}\right)≃\sum_{s}x_{s}\left(t_{i}\right)=D_{T}\left(t_{i}\right)$.

To study the early days of intra-host evolution, we assume negligible back-mutations. Nonetheless, back-mutations and different rates per base can be accounted for by modifying the definition of $\langle d(t_{i})\rangle$ with more fitting substitution models [16-18]. Note that $\langle d(t_{i})\rangle$ differs from intra-population nucleotide diversity, π[19], which is derived from the pairwise comparison of the present genomes at time *t_i_*, whereas $\langle d(t_{i})\rangle$ is derived from comparing those genomes to the original clone at time *t_0_*.

Let$m_{j}\left(t_{i}\right)$ be the number of accumulated polymorphisms at time *t_i _*in sequence *j *since the start of the expansion at time *t_0_*. Assuming $m_{j}\left(t_{i}\right)$ is Poisson distributed with mean ${\bar{\mu }}_{i}l\left(t_{i}-t_{0}\right)$, the log-likelihood of the observed state is $L\left({\bar{\mu }}_{i},t_{0}\right)\approx \sum_{i,j}\left(m_{j}\left(t_{i}\right)\text{log(}{\bar{\mu }}_{i}l\left(t_{i}-t_{0}\right)\text{)}-{\bar{\mu }}_{i}l\left(t_{i}-t_{0}\right)\right)$. In all summations, *i *counts the number of time points, and *j *counts the number of sampled viral clones in *t_i_*. Since the total number of mutations in the population can be counted across the genomes, or equivalently via the frequency of the segregating sites, a crucial observation can be made that $\sum_{j}m_{j}\left(t_{i}\right)=N\left(t_{i}\right)\sum_{s}x_{s}\left(t_{i}\right)=N\left(t_{i}\right)\langle d(t_{i})\rangle$, leading to$L\left({\bar{\mu }}_{i},t_{0}\right)\approx \sum_{i}\left(N\left(t_{i}\right)\langle d(t_{i})\rangle \text{log(}{\bar{\mu }}_{i}l\left(t_{i}-t_{0}\right)\text{)}-N\left(t_{i}\right){\bar{\mu }}_{i}l\left(t_{i}-t_{0}\right)\right)$. Thus, the maximum likelihood estimate (MLE) of the evolutionary rates and the time of the initial clone can be derived from maximizing $L\left({\bar{\mu }}_{i},t_{0}\right)$. In these estimates, *D_T _*or the sum of MAF at *t_i _*are used to approximate $\langle d(t_{i})\rangle$. Maximizing a likelihood, in which there are more parameters than data points without any constraints will lead to over-fitting the data. We follow Sanderson's modeling of a relaxed molecular clock and penalized likelihood approach [20,21], and utilizing a non-parametric regularization term,$W\left({\bar{\mu }}_{i}\right)=\sum_{i}{\left({\bar{\mu }}_{i}-{\bar{\mu }}_{i-1}\right)}^{2}$, we minimize $\text{Ψ}({\bar{\mu }}_{i},t_{0}\text{)}=-L\left({\bar{\mu }}_{i},t_{0}\right)+\lambda W\left({\bar{\mu }}_{i}\right)$, where λ is the smoothing parameter. For very large λ, minimizing  Ψ leads to estimates equal to predictions under a strict molecular clock model. On the other hand, small λ leads to over-fitting the likelihood, and the estimates will be highly affected by small changes in the data. Therefore, an intermediate value of λ should be chosen, so that the estimates follow the data while avoiding numerical artifacts caused by over-fitting. We determine this value by minimizing  Ψ over a range of values for λ and comparing the resulting values of *L *versus those of *W*, by scaling them between 0 and 1. In other words, the maximum *L *is obtained when λ=0 (corresponding to scaled *L *and *W *of 1) and the minimum *W *is obtained when λ→∞ (corresponding to scaled *L *and *W *of 0). We choose the value of λ that results in equally weighted scaled *L *and scaled *W *[22]. For all optimization problems in our method, we employ the non-linear Active Set algorithm [23] as implemented in MATLAB and R. In each optimization, we require $0<{\bar{\mu }}_{i}$ and $t_{0}<t_{1}$.

To calculate standard errors for estimates of $\langle {\bar{\mu }}_{i}\rangle$ and *t_0_*, we generate 1,000 bootstrap sets by permuting the sequences in each dataset. Using each dataset's smoothing parameter, we obtain maximum likelihood estimates for $\langle {\bar{\mu }}_{i}\rangle$ and *t_0_*. The bootstrap estimates are normally distributed and are used to calculate 95% confidence intervals. The presence of purifying selection can be measured through $\omega =\beta \frac{\mu_{non-syn.}}{\mu_{syn.}}$, when it is less than 1. Here, λ is the ratio between the number of synonymous to non-synonymous sites in the genome, which we obtain by randomly mutating the viral genome one million times, assuming equal probability for transition and transversion events.

**Simulated data.**Starting from a single homogenous 10,000 base-long clone, we simulated an exponentially expanding population at 12 time steps. The substitution rate was set at 10^-4 ^substitutions/site per time point in addition to a noise term with a mean of zero and standard deviation of 10^-4^. At each time point, 5,000 sequences were randomly sampled, simulating a typical depth of 5,000x for deep-sequencing. We repeated this procedure 1,000 times.

**Influenza data.** Influenza consensus full-length sequences were obtained from Influenza Virus Resource Database [24] and GISAID [25], selecting H1N1 pandemic strains collected between March 2009 and March 2010. We aligned the sequences of each segment using the MUSCLE algorithm, and further manual curation.

**High-throughput marburgvirus data.**Two separate animal studies provided the samples used in this study. Blood from cynomolgus macaques was collected from NHP therapeutic efficacy trial control animals (saline treated only) on days 8 and 10 of the infection. The viral RNA was extracted and sequenced. We rigorously cleaned the sequence reads to remove systematic errors and identified statistically significant single nucleotide substitutions. The ethics statement and details of the library preparation, sequencing, and variant calling are provided in Supplementary Methods (Additional file 1).

## Results

**Figure 2:**
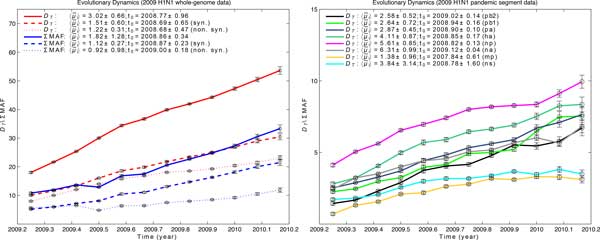
**The maximum likelihood estimates for the 2009 H1N1 influenza pandemic**. **Left: **Whole-genome data estimates based on both MAF and *D_T. _***Right: **Individual segments' estimates based on *D_T_*. (For the MAF-base estimates, see Supplementary Fig. S2, in Additional file 1.) PB2, PB1, and PA encode the RNA polymerase; HA and NA encode the glycoproteins hemagglutinin and neuraminidase; NP, M, and NS segments code the nucleoprotein, matrix proteins and non-structural proteins. Due to structural constraints and small size, the latter three segments accumulate the least number of mutations. Our estimates for the evolutionary rates, the starting time of the expansion, and presence of strong purifying selection (ω = 0.22) corroborated phylogenetic results. The mean evolutionary rate, $\langle {\bar{\mu }}_{i}\rangle$, is in 10^-3 ^substitutions/site/year and *t_0 _*is in days. The standard errors are the 95% confidence intervals via bootstrapping.

**Simulated data.** In a set of 1,000 simulations, the estimates of evolutionary rates between time points captured the expected evolutionary dynamics ($\langle {\bar{\mu }}_{i}\rangle$ = 10^-4 ^substitutions/site per time point), within statistical fluctuations, as shown in Figure 2 (right). In particular, the estimates from *D_T _*found the average of evolutionary rates, $\langle {\bar{\mu }}_{i}\rangle$, to be 0.99 ± 0.22 × 10^-3 ^substitutions/site per time point, and the starting time of the expansion to be at -0.03 ± 0.62. The estimates based on MAF indicated the lower bound of those from *D_T _*(Figure 1).

**The 2009 H1N1 influenza pandemic**. The influenza genome consists of eight single-stranded RNA segments, which code for 10 or more proteins. The novel influenza A virus responsible for the 2009 pandemic was first identified in late March in California and Mexico [26], and spread quickly, as very limited previous immunity to the new strain existed within the human population. Phylogenetic analyses estimated the most recent common ancestor of this strain to have arisen around January 2009 (no earlier than August 2008), and to have evolved with a rate of 3.67 ± 3.05 × 10^-3 ^substitutions/site/year [14,27]. These analyses also identified purifying selection during the pandemic (ω < 1) [15]. The exact genome of the initial virus that infected the human population is not known; however, we approximated a proxy based on the consensus genomes of strains collected early in the expansion (Additional file 2). We found the estimates for the mean of evolutionary rates between time points, $\langle {\bar{\mu }}_{i}\rangle$ and the starting time of the pandemic, *t_0_*, based on both *D_T _*and sum of MAF to be consistent across all segments (Figure 2 (right) and Supplementary Fig. S2, in Additional file 1). As there has been no evidence for reassortment events during the 2009 H1N1 clonal expansion in humans [28], we concatenated the segments and estimated $\langle {\bar{\mu }}_{i}\rangle$ and *t_0 _*using whole-genome data. As shown in Figure 2 (left), the MAF-based estimates for *t_0 _*agreed with those from *D_T_*, and were found to be between November 2008 and January 2009. We also estimated $\langle {\bar{\mu }}_{i}\rangle$ of 1.82 ± 1.28 × 10^-3 ^and 3.02 ± 0.66 × 10^-3 ^substitutions/site/year during the pandemic, according to *D_T _*and sum of MAF, respectively. We also identified a strong purifying selection during this period (ω = 0.22), corroborating results from phylogenetic methods.

**Deep sequencing of marburgvirus from infected NHP.** Marburgvirus, in the *Filoviridae *family, is a single-stranded RNA genome of about 19,000 bases that encodes seven proteins, with an estimated evolutionary rate of 0.1-1.0 × 10^-3 ^substitutions/site/year [29]. Cynomolgus macaque constitutes a commonly used model organism for infection of filoviruses, recapitulating some of the clinical features of infection in humans. Marburgvirus causes hemorrhagic fevers in humans and NHP, who typically succumb to the infection in 8-12 days.

**Figure 3:**
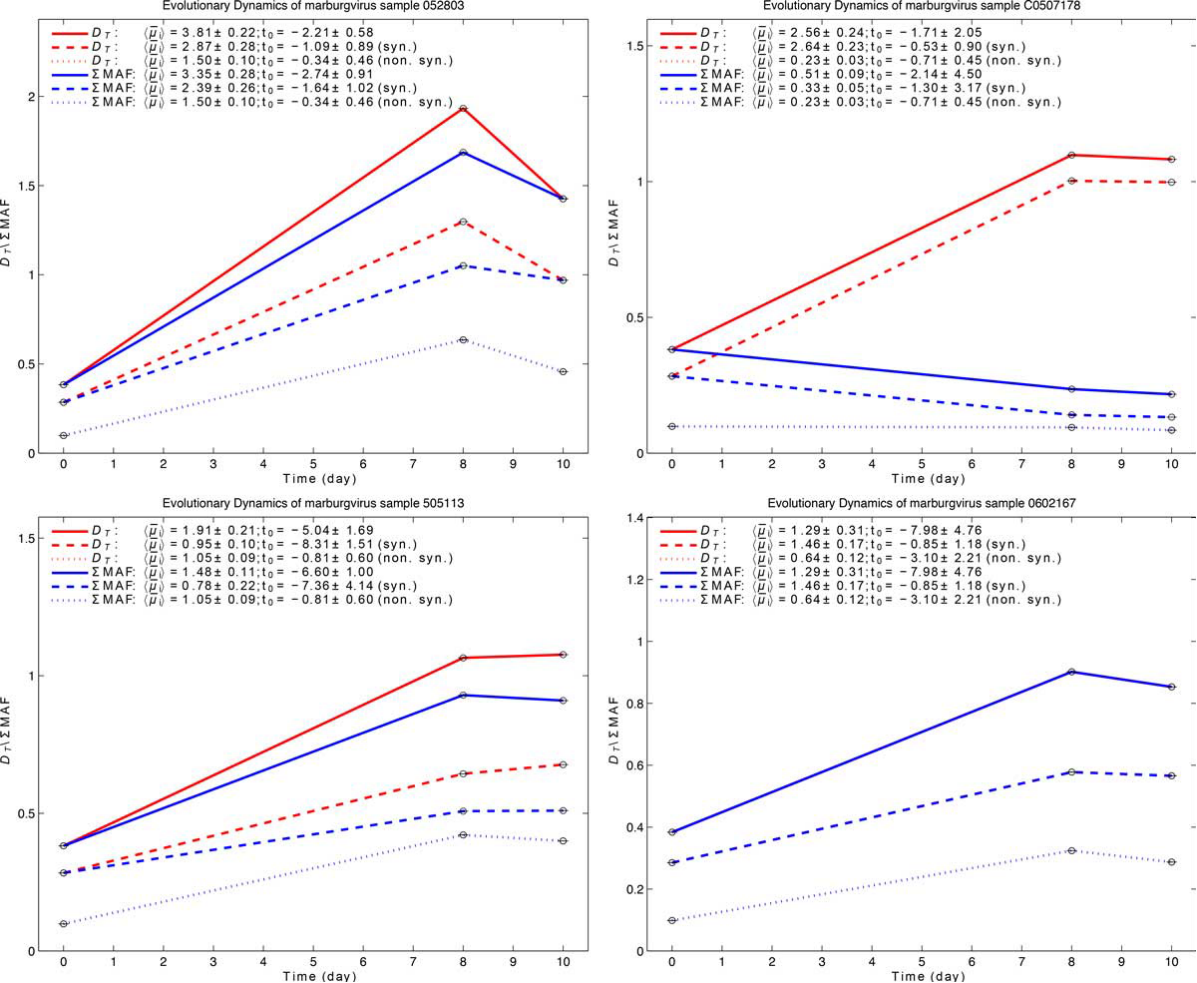
**The maximum likelihood estimates for four marburgvirus samples from infected NHP**. We found the estimated intra-host evolutionary rates for non-synonymous substitutions to be in similar range. In three samples, MAF-based and *D_T _*measures differed for synonymous substitutions, due to increases in frequency of a single allele. In the fourth sample, MAF-based and *D_T _*measures were identical and overlapped. The mean evolutionary rate, $\langle {\bar{\mu }}_{i}\rangle$, is in 10^-3 ^substitutions/site/year and *t_0 _*is in days. The standard errors are the 95% confidence intervals via bootstrapping.

Working from an existing study of cynomolgus macaques infected with a Musoke strain marburgvirus, we utilized deep sequencing data (coverage depth >10,000x) of viral RNA collected at different time points from four samples (505113, 052803, C0507178, and 0602167, as shown in Supplementary Table S1, in Additional file 1). We obtained frequency estimates as low as 0.05% for an average of 60 variants per sample (range 26 to 110, as listed in Supplementary Tables S2 and S4, in Additional file 1 and Additional file 3 respectively). We found ~3.5 times more transitions than transversions across samples (Supplementary Table S3, in Additional file 1), and observed a very homogenous viral population in the challenge stock (day 0) and a subsequent increase in viral diversity over time *in vivo *in all four individual experiments (Figure 3). The four independent analyses showed similar results, 1) an increasing genomic diversity with $\langle {\bar{\mu }}_{i}\rangle$ of 0.23-1.50 × 10^-3 ^substitutions/site/year for non-synonymous substitutions and 1.29-3.81 × 10^-3 ^for all substitutions; 2) 2-8 days to convergence with the reference, approximately the amount of time spent propagating the virus after it was originally sequenced [30].

Acknowledging the caveat that each of the four samples went through different host-specific immune responses, we combined the data and obtained estimate for $\langle {\bar{\mu }}_{i}\rangle$ to be 2.11 ± 1.76 × 10^-3 ^substitutions/site/year for non-synonymous substitutions and 2.95 ± 0.48 × 10^-3 ^for all substitutions (Supplementary Fig. S3, in Additional file 1). We also identified strong purifying selection (ω = 0.43).

## Discussion

We have proposed two measures of genetic diversity, derived independently of phasing information: 1) total divergence, *D_T_*, the sum of frequencies of diverging alleles from the original clone, and 2) the sum of minimal allele frequencies (MAF) at segregating sites. Our methodology is robust to recombination or reassortment events within a clonal population because such evolutionary processes do not affect our measures of genetic diversity. Since the numbers of sites with diverging alleles in a sampled population, acquired within the first few days of an acute infection or the early months of an outbreak, are much smaller than the length of the viral genome, the assumption that their distribution between two time points can be approximated with Poisson distributions holds. Assuming negligible positive selection and back-mutations, *D_T_*, increases over time by definition; thus, it measures divergence from the seed of the expansion. On the other hand, the sum of MAF measures population diversity at a particular moment in time. Therefore, strong differences between the two measures indicate deviations from neutral evolution, selection, or bottlenecks. Our approach is particularly novel in its independence from an assumed growth model or previously published evolutionary rates, used in similar applications to intra-host data [5]. Since we assume that the number of segregating sites is much smaller than the length of the viral genome, and that the infection starts by a genetically uniform population, our method is applicable to lytic viruses, and cannot be applied to integrating or lysogenic viruses. Based on these measures, we followed a penalized maximum likelihood approach and a model of relaxed molecular clock [20,21], and were able to estimate the starting point in time and evolutionary rate of clonal expansions.

To evaluate our method with well-characterized examples of clonal expansion, we calibrated it with a set of simulated sequences following a relaxed molecular clock model, and obtained estimates that capture the evolutionary parameters of the generating model. We found the estimates obtained from sum of MAF to be the lower bound of those from total divergence. With the purpose of comparing and validating our methodology with standard phylogenetic techniques, we utilized phased whole-genome sequence data from the 2009 influenza pandemic. Limiting the data to the H1N1 isolates collected within the first year after the start of the pandemic, our estimates for the mean evolutionary rate, the starting time of the expansion, and presence of strong purifying selection corroborated with phylogenetic results [14,15,27]

The novelty and most important application of our method is in analyzing unphased temporal data to which phylogenetic methods cannot be applied. During the course of an acute infection, the diversification of the viral population is not reflected in the consensus sequence, as most changes are minor, rare variants. To study viral intra-host diversity, we employed genomic data obtained from high-throughput ultra-deep sequencing of marburgvirus from four infected NHP, sampled at days 8 and 10 of the infection. The results showed consistent increases in viral diversity and the starting time of the intra-host expansion was found in agreement with the experimental setup [30]. MAF-based diversity measures for non-synonymous substitutions in three of the infected NHP presented extremely good approximations for *D_T_*, which is especially important when the seed of a clonal expansion is not known (Figure 3). In particular, we found the estimated intra-host evolutionary rates for non-synonymous substitutions to be in similar range but higher than those reported from inter-host phylogenetic analysis [29]. Combining the data from four samples corroborated with individual analyses, and the ratio of non-synonymous to synonymous substitutions rates indicated similar strong purifying selection to inter-host transmission of marburgvirus [31].

In three samples, MAF-based and *D_T _*diversity measures differed for synonymous substitutions, due to increases in frequency of a single allele (E142E) in the *L *gene. This allele increased from 6% in the seed stock to 62% (052803), 57% (505113), and 92% (C0507178) on day 8. The frequencies on day 10 were similar to those on day 8, except in one sample (052803), in which it fell to 31%. In one sample (0602167) the frequency of this allele was found to be 23% on both day 8 and day 10, not affecting MAF. Synonymous mutations have been shown to contribute to viral fitness in other viruses [4], and despite the fact that this allele did not alter the coding of the L protein, the presence of a selection pressure that leads to increases in its frequency cannot be ruled out.

## Conclusion

As technology progresses, deep sequencing of temporal samples is becoming more readily available; however, due to missing phasing information, the application of standard phylogenetic methods to these data sources is limited. The measures of diversity defined in this manuscript present a distinct advantage over methods based on consensus sequences, specifically because of their power to analyze genomic diversification within days of an infection. This method is an ideal tool to pinpoint the time of infection, to estimate the evolutionary rate within a host, and to identify early markers of selection, in the course of an acute infection.

## List of abbreviations

Non-human primates (NHP). Minimal allele frequencies (MAF).

## Competing interests

The authors declare that they have no competing interests.

## Authors' contributions

HK designed, developed, and validated the mathematical model; ZC analyzed the influenza dataset, JK analyzed the marburgvirus dataset, JC and VT contributed to the mathematical model, EN, TW, PI, and SB contributed to and GP directed the analysis of the high-throughput data, RR designed the study and directed research. All authors wrote and edited the manuscript.

## Supplementary Material

Additional file 1 Click here for file

Additional file 2 Click here for file

Additional file 3 Click here for file

## Declaration

This work was funded by the Defense Threat Reduction Agency (DTRA) Project No. 1899628 and DTRA grant W81XWH-13-2-0029. The publication of this work was funded by a grant from the Geneva Foundation (HDTRA1-14-1-0016). The funders had no role in the design, collection, analysis, and interpretation of data.

This article has been published as part of *BMC Genomics *Volume 15 Supplement 6, 2014: Proceedings of the Twelfth Annual Research in Computational Molecular Biology (RECOMB) Satellite Workshop on Comparative Genomics. The full contents of the supplement are available online at http://www.biomedcentral.com/bmcgenomics/supplements/15/S6.
