# Supplementary material for: Viral diversity and clonal evolution from unphased genomic data
Source: BMC Genomics. 2014 Oct 17;15(Suppl 6):S17. doi: 10.1186/1471-2164-15-S6-S17 (PMC4240099; doi:10.1186/1471-2164-15-S6-S17)
Supplement: Additional file 1 [file 1471-2164-15-S6-S17-S1.pdf]

Supplementary Methods

Ethics statement. Research was conducted under an IACUC approved protocol AP-11-027 in compliance with the Animal Welfare Act, PHS Policy, and other Federal statutes and regulations relating to animals and experiments involving animals. The facility where this research was conducted is accredited by the Association for Assessment and Accreditation of Laboratory Animal Care, International and adheres to principles stated in the Guide for the Care and Use of Laboratory Animals, National Research Council, 2011. The Institutional Animal Care and Use Committee of the United States Army Medical Research Institute of Infectious Diseases approved these studies. Animals were individually housed in stainless steel cages and were provided food and water ad libitum. Animal rooms were maintained on a 12-h light/dark cycle and the animals were provided toy and fruit environmental enrichments. Animals were monitored at least twice daily for signs of distress. Buprenorphine was administered to animals displaying clinical signs of discomfort and meloxicam was administered to animals exhibiting elevated body temperature. Euthanasia was performed to minimize pain and distress by intravenous administration of sodium pentobarbital.

RNA extraction and high-throughput sequencing. Two separate animal studies conducted at USAMRIID (AP-09-033 and AP-09-029) provided the samples used in this study. Blood from cynomolgus macaques was collected from NHP therapeutic efficacy trial control animals (saline treated only) on days 8 and 10 of the infection. Viral RNA was extracted using Trizol LS (Invitrogen, Carlsbad, CA). First-strand synthesis was performed with the Superscript III first-strand synthesis system (Life Technologies/Invitrogen, Carlsbad, CA) and specific primers were used to amplify the viral genome. After purification with the MinElute PCR purification kit (QIAgen, Valencia, CA), PCR products were fragmented using the Covaris S2 instrument (Covaris, Woburn, MA). Libraries were prepared with the Illumina TruSeq DNA sample Preparation kit (Illumina, San Diego, CA), according to the manufacturer's protocol. The libraries were evaluated for quality using the Agilent 2100 Bioanalyzer (Agilent, Santa Clara, CA). After

measurement by real-time PCR with the KAPA qPCR Kit (Kapa Biosystems, Woburn, MA), libraries were diluted to 10 nM. Cluster amplification was performed on the Illumina cBot and libraries were sequenced on the Illumina GAIIx using the 76 bp paired-end format.

The high-throughput sequence reads' preprocessing was completed using a combination of PRINSEQ-lite [1] and in-house cleaning scripts. We initially restricted the reads to those having an average index sequence phred quality score > 30. We then used an in-house cleaning tool, readCleaner, which is a BLAST based tool for the identification and removal of primer sequences, sequencer or PCR based chimeric reads, non-viral sequence prior to reference alignment, and any alignment errors that occur at the end of a read. We specifically removed primer sequences for the ~10-12% error rates introduced during oligo synthesis (4% substitution and 8% early termination errors resulting in indels). To remove any possibility of a sequence being improperly trimmed at a junction site, we removed the read prior to alignment. Improperly phased and poor quality base calls are more common at the end of a read and were removed from the alignment. This filter had the effect of making more conservative calls at the site of the mutation or SNP but also removed any unfiltered tag and adaptor sequence prior to alignment. We also performed a mandatory 5 bp 5' trim and a 15 bp 3' trim to remove any untrimmed primer or random hexamer and generally improve quality and then removed any remaining mate pair singletons. Finally, the reads in the clean dataset were mapped to the reference sequence for marburgvirus strain Musoke (GenBank: DQ217792). Reference alignments were analyzed for 3' error rates and if needed, reads were subjected to further end trimming and subsequent reassembly. Viral assemblies were completed in Lasergene nGen (http://www.dnastar.com/t-nextgen-seqman-ngen.aspx).

Identification of variants. The rigorous cleaning of the sequence data removed systematic errors, in particular the substantial bias towards transversions [2], instead of the commonly observed transitions in organisms [3]. We limited the variant calling to the coding regions, covered at >85% with depths >700x in all temporal samples of each case. We used the SAVI

(Statistical Algorithm for Variant Identification) algorithm [4] to identify statistically significant variants. SAVI constructed empirical priors for the distribution of variant frequencies in each sample, from which we obtained a corresponding high-credibility interval for the frequency of a particular allele. To obtain estimates for alleles with frequencies as low as 0.05%, we chose logarithmically spaced precision for the priors and posteriors. Alleles with posterior probability less than 10^{-3} were considered to be present in the sample.

References

1. Schmieder R, Edwards R: **Quality control and preprocessing of metagenomic datasets**. *Bioinformatics* 2011, **27**(6):863-864.
2. Dohm JC, Lottaz C, Borodina T, Himmelbauer H: **Substantial biases in ultra-short read data sets from high-throughput DNA sequencing**. *Nucleic acids research* 2008, **36**(16):e105.
3. Wakeley J: **The excess of transitions among nucleotide substitutions: new methods of estimating transition bias underscore its significance**. *Trends in ecology & evolution* 1996, **11**(4):158-162.
4. Trifonov V, Pasqualucci L, Tiacci E, Falini B, Rabadan R: **SAVI: a statistical algorithm for variant frequency identification**. *BMC Systems Biology* 2013, **7(Suppl 2)**:S2.

Supplementary Figures

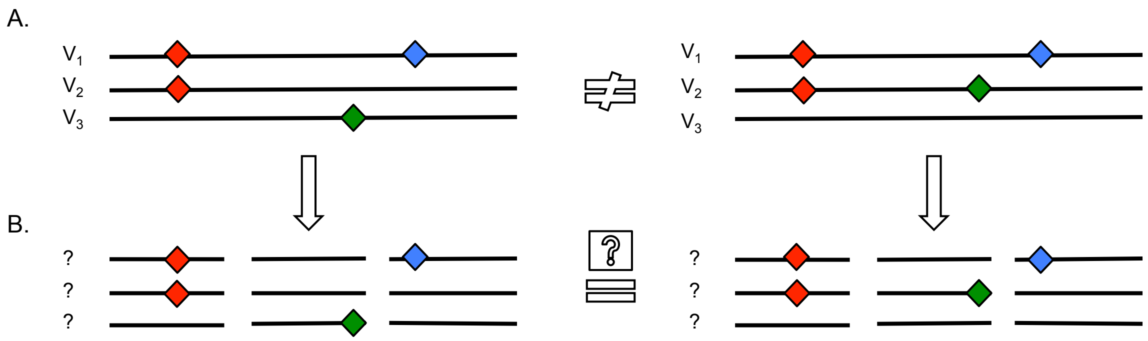

Figure S1 – Information on coexistence of polymorphisms in each clone in phased versus unphased data. Panel A depicts two scenarios where sequence data is phased, and thus the knowledge of mutation linkage with respect to genome is available. Panel B depicts the outcome of a high-throughput sequencing process in which due to short read-length, the information of mutation linkage with respect to individual genomes is lost.

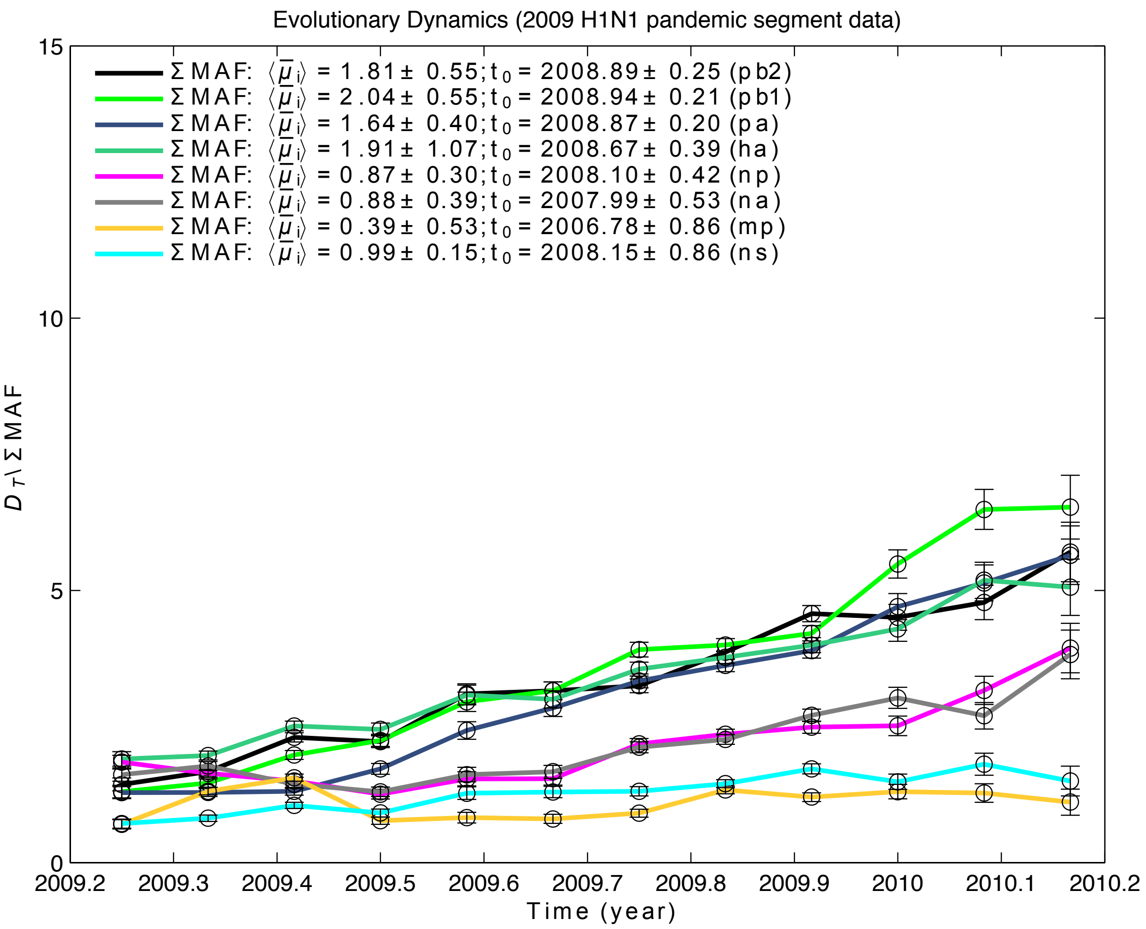

Figure S2 – The maximum likelihood estimates for the 2009 H1N1 influenza pandemic based on MAF. The mean evolutionary rate, $\langle\bar{\mu}_i\rangle$, is given in 10^{-3} substitutions/site/year and t_0 is in years. The standard errors are derived from 95% confidence intervals via bootstrapping.

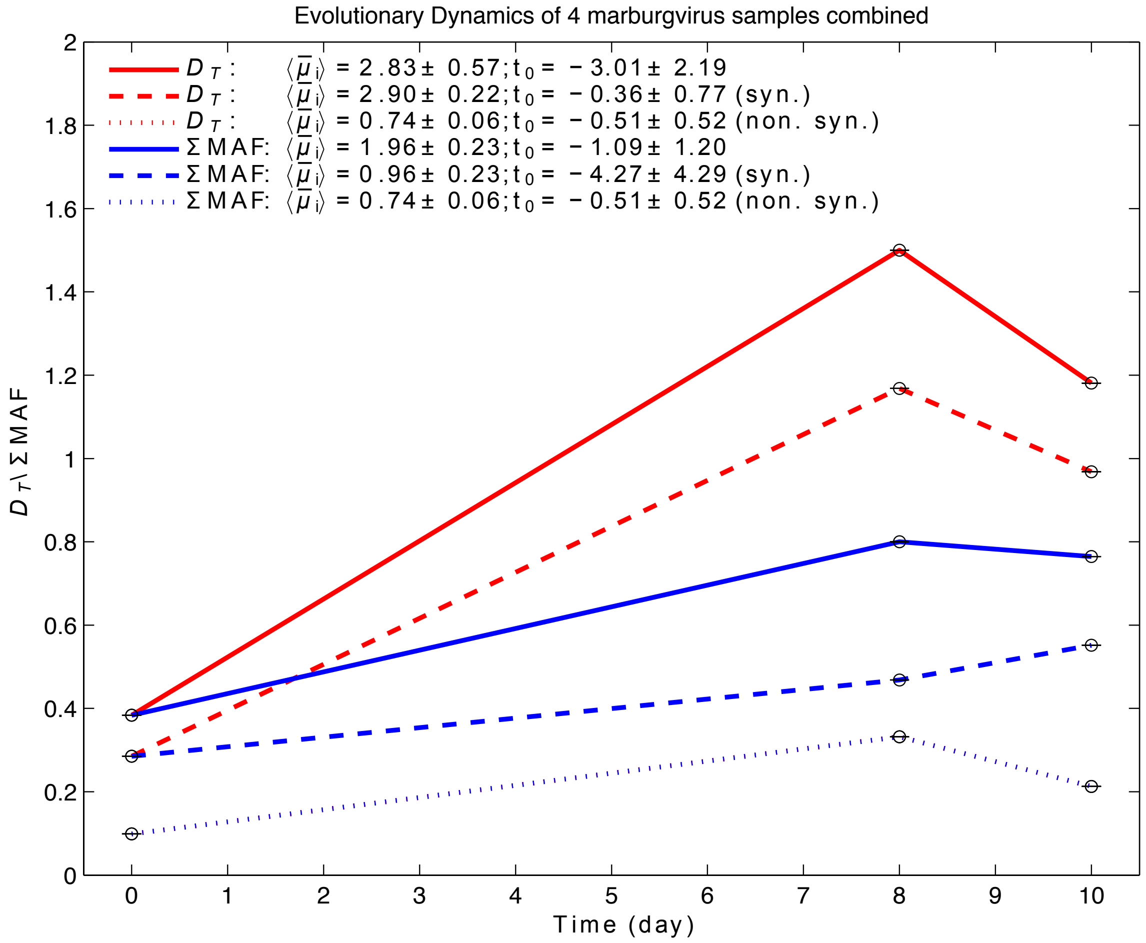

Figure S3 – The maximum likelihood estimates for combined data from four marburgvirus samples. For non-synonymous variants, MAF-based and D_T measures were identical and overlapped. The mean evolutionary rate, $\langle\bar{\mu}_i\rangle$**,** is given in 10^{-3} substitutions/site/year and t_0 is in days. The standard errors are derived from 95% confidence intervals via bootstrapping.

Supplementary Tables

Table S1 – The mean sequencing depth of samples collected from blood from cynomolgus macaques on each day. The seed of the infection sequenced at the mean depth of 30,611. The coding region of marburgvirus was 100% covered across all time points.

Sample	Day 8	Day 10
505113	7481	15696
52803	12647	10500
C0507178	14247	14155
602167	11585	11124

Table S2 – Number of substitutions in the coding region of marburgvirus samples across all time points.

Sample	Day of collection	All Substitutions	Non-synonymous substitutions
Seed	0	94	52
505113	8	42	28
	10	76	48
52803	8	110	63
	10	72	40
C0507178	8	26	18
	10	28	19
602167	8	44	21
	10	40	21

Table S3 – Number and types of mutations across all samples. We found ~3.5 times more transitions (top 4 rows) than transversions.

Mutation	Count
T > C	36
G > A	32
A > G	30
C > T	27
G > T	13
C > A	10
A > C	4
G > C	3
T > G	3
A > T	2
T > A	1
